# Can Women Farm?

**Published:** 1894-06

**Authors:** 


### Can Women Farm? 56,800 Women are Farming in America.

America had in 1890, 2,700,000 breadwinning women and girls working outside of their own homes. There were 110 lawyers, 165 ministers, 320 authors, 588 journalists, 20,061 artists, 2,136 architects, chemists, pharmacists, 2,106 stock raisers and ranchers, 5,135 government clerks, 2,538 physicians and surgeons, 13,182 professional musicians, 56,800 farmers and planters, 21,071 clerks and bookkeepers, 14,465 heads of commerical houses, 155,000 public school teachers (based on the census of 1880).—*Woman's Journal.*

